# Gene regulatory networks in neural cell fate acquisition from genome-wide chromatin association of Geminin and Zic1

**DOI:** 10.1038/srep37412

**Published:** 2016-11-24

**Authors:** Savita Sankar, Dhananjay Yellajoshyula, Bo Zhang, Bryan Teets, Nicole Rockweiler, Kristen L. Kroll

**Affiliations:** 1 Department of Developmental Biology, Washington University School of Medicine, 660 S. Euclid Avenue, Saint Louis, MO 63110, USA; 2 Department of Genetics, Washington University School of Medicine, 660 S. Euclid Avenue, Saint Louis, MO 63110, USA.

## Abstract

Neural cell fate acquisition is mediated by transcription factors expressed in nascent neuroectoderm, including Geminin and members of the Zic transcription factor family. However, regulatory networks through which this occurs are not well defined. Here, we identified Geminin-associated chromatin locations in embryonic stem cells and Geminin- and Zic1-associated locations during neural fate acquisition at a genome-wide level. We determined how Geminin deficiency affected histone acetylation at gene promoters during this process. We integrated these data to demonstrate that Geminin associates with and promotes histone acetylation at neurodevelopmental genes, while Geminin and Zic1 bind a shared gene subset. Geminin- and Zic1-associated genes exhibit embryonic nervous system-enriched expression and encode other regulators of neural development. Both Geminin and Zic1-associated peaks are enriched for Zic1 consensus binding motifs, while Zic1-bound peaks are also enriched for Sox3 motifs, suggesting co-regulatory potential. Accordingly, we found that Geminin and Zic1 could cooperatively activate the expression of several shared targets encoding transcription factors that control neurogenesis, neural plate patterning, and neuronal differentiation. We used these data to construct gene regulatory networks underlying neural fate acquisition. Establishment of this molecular program in nascent neuroectoderm directly links early neural cell fate acquisition with regulatory control of later neurodevelopment.

Formation of the complex vertebrate nervous system is initiated when pluripotent cells of the early embryo acquire a neural fate. This process accompanies induction and patterning of the three germ layers during gastrulation. In the mammalian embryo, uncommitted epiblast cells move through the primitive streak and develop into mesodermal and endodermal derivatives. Anterior epiblast cells that do not enter the primitive streak instead develop into ectoderm, some of which is induced to form the future neural plate^1^,^2^. Growth factor signaling regulates this process, with BMP, Nodal, and Wnt signaling inducing mesendoderm formation and patterning, while regionalized inhibition of these growth factor signals induces neuroectodermal cell fate acquisition^3^,^4^.

Mouse embryonic stem (ES) cells, derived from the inner cell mass of the preimplantation blastocyst embryo, provide a useful model for understanding neuroectodermal cell fate acquisition at the molecular level. As occurs *in vivo*, blockade of growth factor signaling initiates neuroectodermal fate acquisition of ES cells^3^,^4^,^5^,^6^,^7^. This involves exit from the pluripotent state and expression in nascent neuroectoderm of a suite of neural transcription factors (nTFs). These nTFs serve as early, cell intrinsic regulators of neural fate acquisition^8^,^9^,^10^. They activate and maintain gene expression programs that stabilize neural fate acquisition and their activity precedes later neurodevelopmental events, including neural plate regionalization, neurogenesis, and the transition from neural progenitor expansion and maintenance to neuronal differentiation.

The nTFs include several Zic zinc-finger and SoxB1 class transcription factors^8^,^9^,^10^,^11^,^12^,^13^ and the nuclear regulator Geminin (Gmnn). Gmnn controls gene expression through interactions with chromatin modifying complexes and transcription factors^8^,^9^,^10^,^14^,^15^,^16^,^17^ and is also one of several cellular inhibitors of genome re-replication within each cell cycle^18^. During early embryogenesis in Xenopus, the nTFs are broadly expressed in nascent neuroectoderm as an early response to neural induction^8^,^9^,^10^,^19^,^20^. Cross-regulatory relationships between Gmnn, Sox1-3, and Zic1-3 cooperatively promote neural fate acquisition and control expression of later acting transcription factors, including the Iroquois homeodomain transcription factors, which regulate regional neural plate patterning^8^,^9^,^10^. Many of these nTFs also have conserved expression and roles in regulating mammalian neural development^8^,^9^,^10^,^11^,^12^,^13^. In the mouse, Gmnn and Sox2 are expressed in pluripotent embryonic cells of the inner cell mass^21^,^22^, with expression becoming enriched in nascent neuroectoderm by late gastrulation^23^,^24^. This expression overlaps that of Sox3 and Zic1, which are also broadly expressed in nascent neuroectoderm by embryonic day 7.5–8 (E7.5-8)^24^,^25^,^26^. However, regulatory networks through which Gmnn and several of the other nTFs control neural cell fate acquisition remain largely undefined.

Geminin plays essential roles in early embryogenesis. In Xenopus, Gmnn promotes neuroectoderm formation at the expense of non-neural tissues (epidermis, mesendoderm)^14^,^27^. Gmnn null mice exhibit early lethality prior to neural tissue formation, failing to form the inner cell mass, while all cells instead develop as extraembryonic trophectoderm^21^. Conditional Gmnn knockout in mice also demonstrates requirements in early neural development. Gmnn deficiency in the epiblast from E5.5, or restricted to the rostral or caudal neural plate from ~E8.0, results in cranial or caudal neural tube defects, with diminished expression of genes that control neural plate patterning, neurogenesis, and neuronal differentiation^28^. Manipulation of Gmnn levels during directed ES differentiation likewise demonstrates roles for Gmnn in promoting neural cell fate acquisition and in repressing mesendodermal fate acquisition^29^,^30^.

Geminin can regulate gene expression through direct interactions with both chromatin modifying complexes and homeodomain transcription factors^15^,^16^,^17^,^31^. During neural fate acquisition of ES cells, Gmnn promotes neural gene expression by maintaining chromatin in an acetylated and accessible state, while Gmnn can also rapidly enhance chromatin acetylation and accessibility *in vitro* when recombinant Gmnn is combined with ES nuclear extract and chromatin^29^. However, genome-wide profiles of Gmnn association and Gmnn-dependent histone acetylation, through which Gmnn controls neural fate acquisition, had not been defined.

Here, we endeavored to understand how Geminin controls neural cell fate through interactions with chromatin and used these data to define gene regulatory networks underlying neural fate acquisition. We used chromatin immunoprecipitation (ChIP) and next generation sequencing (ChIP-seq) to define Gmnn-associated chromatin locations in ES cells and ES-derived neuroectoderm (NE) at a genome-wide level. We compared these Gmnn-associated gene profiles with effects of Gmnn deficiency on histone acetylation of promoters during neural fate acquisition. During this work, we found that Gmnn associates with the gene encoding Zic1 both in ES cells and in NE. Therefore, we also defined Zic1-associated genes in NE by ChIP-seq and compared these data. Gmnn and Zic1 preferentially bind genes that have embryonic central nervous system-enriched expression *in vivo* and Gmnn and Zic1 singly or cooperatively promote the expression of genes controlling later neural development. We used the shared set of Gmnn- and Zic1-associated transcription factors to construct gene regulatory networks underlying neural cell fate acquisition. This work defines unique and shared targets of Gmnn and Zic1, which act with other nTFs to link transcriptional control of early neural fate acquisition with activation of gene expression programs driving several aspects of later neural development.

## Results

### Genome-wide association of Geminin and Zic1 during neural cell fate acquisition

**Figure 1:**
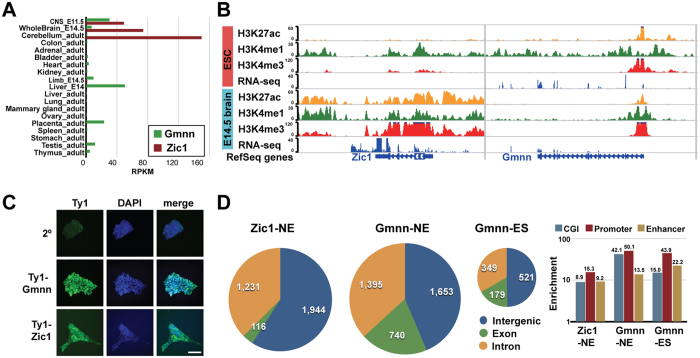
Genome-wide binding profiles for Gmnn and Zic1 during neural cell fate acquisition. (**A,B**) In embryonic brain and/or CNS tissues, Gmnn and Zic1 genes have (**A**) enriched expression and (**B**) enrichment for histone modifications associated with transcriptional activity (WashU Epigenome browser view; http://epigenomegateway.wustl.edu/). (**C**) Clonal ES cell lines were generated with knock-in of a 3X Ty1 epitope tag at the Gmnn or Zic1 genes. Ty1 immunocytochemistry detects expression of Ty1-Gmnn in ES cells and of Ty1-Zic1 in ES-derived neuroectoderm. Scale bar = 50 μm. (**D**) Genomic locations of Gmnn and Zic1 associated peaks, defined by ChIP-seq analysis, and enrichment for genome features (see Methods).

Examination of Geminin and Zic1 expression across embryonic and adult tissues in the mouse revealed strong expression in embryonic central nervous system (CNS) tissue (Fig. 1A; Supplementary Fig. S1). Gmnn expression was highest in the E11.5 CNS but was substantially diminished in E14.5 brain, while adult tissues including cerebellum had low Gmnn expression levels (Fig. 1A). Zic1 was also expressed in the embryonic CNS. However, unlike Gmnn, its expression levels increased from E11.5 through adult in the neural tissues examined (Fig. 1A). Consistent with their CNS-enriched expression, histone modifications associated with transcriptional activity (histone H3 lysine 27 acetylation (H3K27ac) and lysine 4 tri-methylation (H3K4me3)) were apparent at the Gmnn and Zic1 genes in embryonic brain (Fig. 1B).

To define Geminin- and Zic1-associated chromatin regions during neural fate acquisition at a genomewide level, we initially performed ChIP followed by microarray analysis (ChIP-chip) to assess Gmnn promoter occupancy in ES cells and ES cell-derived neuroectoderm. As available Gmnn and Zic1 polyclonal antibodies showed variable, lot-dependent performance in ChIP applications, we also used CRISPR-mediated genome engineering for knock-in of a Ty1 epitope tag at the endogenous Gmnn or Zic1 locus. This enabled genome-wide analysis of Gmnn- and Zic-associated chromatin regions by ChIP-seq. Clonal mouse ES lines were generated that expressed amino-terminal tagged Ty1-Gmnn or Ty1-Zic1, under control of each endogenous locus and its regulatory sequences. ES clones with the correct targeting events were selected by genomic DNA and mRNA analyses (Supplementary Fig. S2) and Ty1-Gmnn or Ty1-Zic1 protein expression in ES cells and NE was confirmed by immunocytochemistry (Fig. 1C).

Two independent, clonal ES lines each for Ty1-Geminin and Ty1-Zic1 were used to perform ChIP-seq analysis for Gmnn in ES cells and for Gmnn and Zic1 in NE. ChIP-seq read depth and Gmnn- and Zic1-associated peaks and nearest transcription start sites are in the Supplementary Material (Supplementary Fig. S3 and Tables S1–S3). By combining peaks identified for Gmnn in both ChIP-chip and ChIP-seq experiments, we identified 1049 Gmnn peaks in ES cells, and 3788 Gmnn peaks and 3291 Zic1 peaks in NE (Fig. 1D). Gmnn- and Zic1-associated peaks were detected predominantly in intergenic and intronic regions, and were enriched in regulatory elements, especially gene promoters (Fig. 1D).

**Figure 2:**
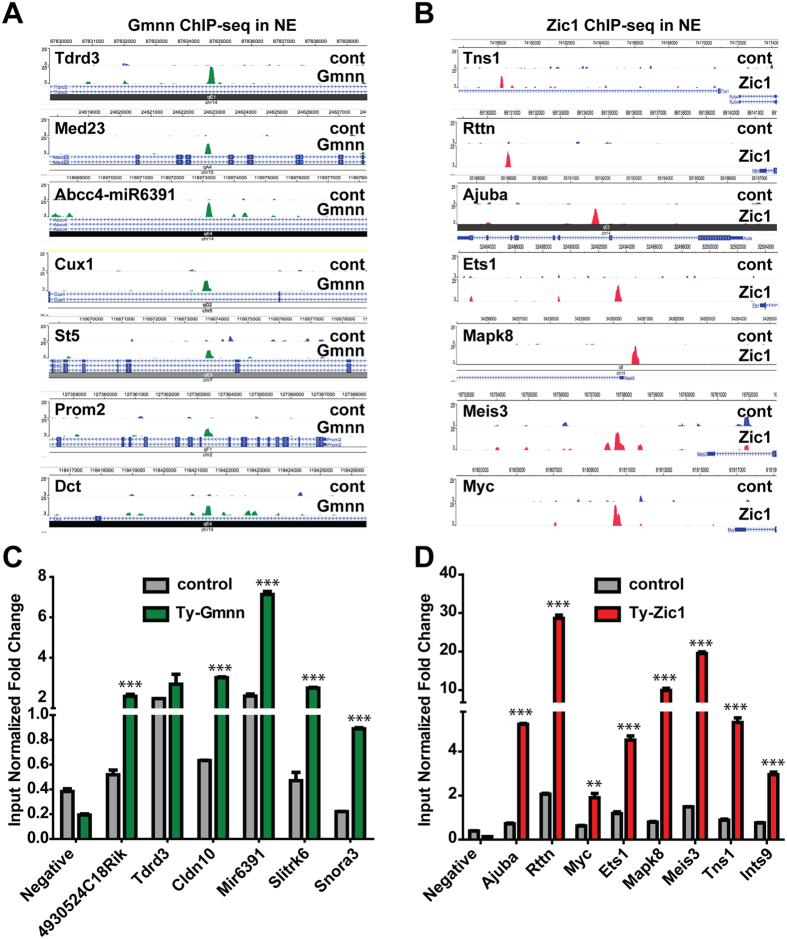
ChIP qPCR testing of peaks obtained from Gmnn and Zic1 ChIP-seq in NE. (**A,B**) Examples of Gmnn and Zic1 ChIP-seq peak density in neuroectoderm (NE). (**C,D**) ChIP-seq validation for Gmnn and Zic1 ChIP-seq. Peak enrichment was assessed using ChIP qPCR with the Ty1 antibody after 3 days of NE fate acquisition in Ty-Gmnn or Ty-Zic1 expressing knock-in ES clonal cell lines. ChIP primers are in Supplementary Table S5. Control (cont) = Ty1 ChIP, performed using the ES-E14TG2a cell line that does not have Ty1-tagged Gmnn or Zic1 knock-in and was differentiated into NE in parallel with experimental samples. Negative = mouse negative control ChIP primer set1 from Active Motif. p-values (student’s t-test) are: *** =< 0.001, ** =< 0.01. Error bars represent standard deviation for a representative qPCR performed in triplicate.

We visualized the ChIP-seq tracks for Geminin and Zic1 in NE (Fig. 2A,B) and used ChIP-qPCR to validate Gmnn and Zic1 association with a subset of these peaks (Fig. 2C,D). We also used ChIP-qPCR to test Gmnn association with peaks in ES cells, focusing on peaks predicted to be Gmnn associated in both ES cells and NE (Supplementary Fig. S4A,B). In addition, for a subset of Gmnn-associated peaks defined by ChIP-chip analysis in ES, we performed ChIP qPCR with both the Gmnn antibody used for ChIP-chip and the Ty1 antibody in the Ty1-Gmnn knock-in ES line (Supplementary Fig. S4C,D), obtaining Gmnn enrichment at these peaks with both experimental approaches. Therefore, we predict that these data represent most high confidence Gmnn-associated chromatin regions in ES cells and NE.

### Geminin associated genes have embryonic CNS-enriched expression and undergo Geminin-dependent histone acetylation during neural fate acquisition

**Figure 3:**
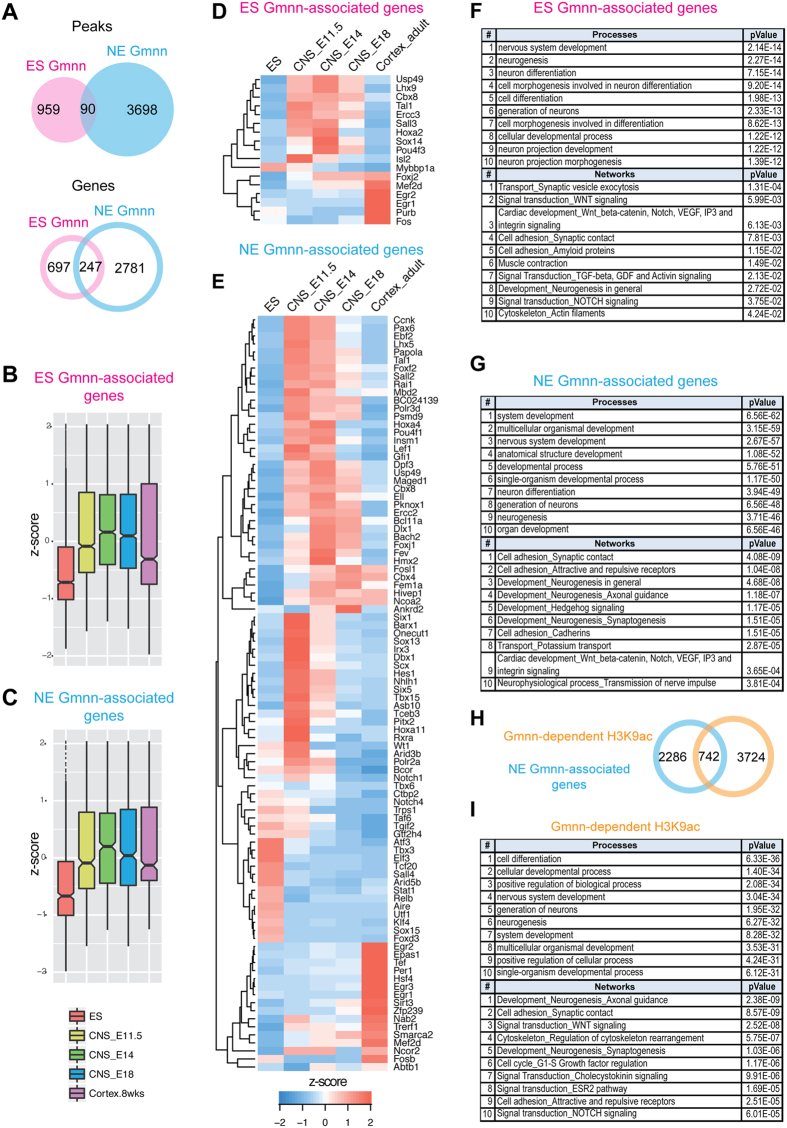
Gmnn-associated genes and genes that undergo Gmnn-dependent histone acetylation exhibit embryonic CNS-enriched expression and neurodevelopmental function. (**A**) Overlap between Gmnn-bound peaks and associated genes in ES cells and in NE. (**B–E**) Gene expression levels in ES cells and in embryonic and adult CNS were (**B,C**) defined for all Gmnn-associated genes and expressed as a z-score or (**D,E**) defined for Gmnn-associated transcription factors and subjected to hierarchical clustering. (**F,G**) GO term enrichment for Gmnn-associated genes (in ES and in NE). Subset shown here had at least two-fold enriched expression in the E14 CNS versus ES cells. Additional GO analysis is in Supplementary Fig. S5. (**H**) Genes that exhibit Gmnn-dependent histone acetylation are compared to those bound by Gmnn in NE, and (**I**) GO analysis of the subset of genes with Gmnn-dependent acetylation that has E14 CNS-enriched expression was defined as in (**F,G**). p-value for A, H (Chi-square test with Yates’ correction) <2.2 × 10^−16^.

Genome-wide profiles of Geminin-associated chromatin were defined for both undifferentiated ES cells and after three days of neuroectodermal cell fate acquisition *in vitro*, as described above. Bound peaks were mapped to the nearest transcription start sites to define gene sets associated with Gmnn in ES and NE. Interestingly, one quarter (247; 26.1%) of the Gmnn associated genes in ES cells were still associated with Gmnn in NE (Fig. 3A). Of these genes, 90 had consistent Gmnn peak locations both in ES cells and during NE fate acquisition, while at the other genes Gmnn associated with a different peak within or near the same gene in ES versus NE (Fig. 3A).

To understand the role of these Geminin-associated genes, we compared each gene set with its expression levels in ES cells and in embryonic CNS tissues (E11.5/14.5/18) or adult cortex. Gmnn-associated genes in both ES and in NE exhibited CNS-enriched expression (Fig. 3B,C). We likewise found that many Gmnn-associated transcription factors in ES cells and NE were most highly expressed in embryonic (E11.5/E14) CNS (Fig. 3D,E).

To determine biological functions of these Geminin associated genes, we used MetaCore (Thomson Reuters) to analyze enriched gene ontology (GO) terms. Gmnn-associated genes in both ES cells and in NE were enriched for genes involved in development and nervous system development (Fig. 3F,G, Supplementary Fig. S5). Subsets of Gmnn-bound genes with embryonic CNS-enriched expression associated with additional GO terms related to early neural development (e.g. neurogenesis, neuron differentiation, synaptogenesis) (Fig. 3F,G). Together, these data indicate that both in ES cells and in NE, Gmnn associates predominantly with embryonic CNS-enriched genes involved in several aspects of neural development.

**Figure 4:**
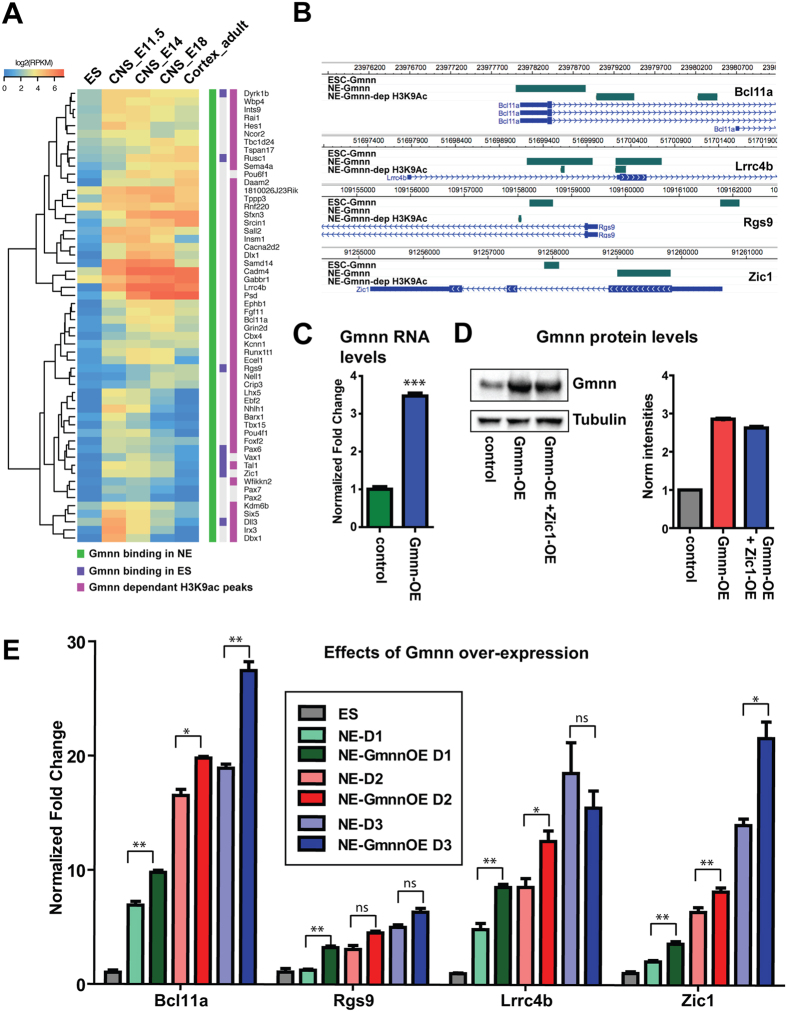
Geminin over-expression during neural cell fate acquisition promotes the expression of Gmnn-associated genes with Gmnn-dependent histone acetylation. (**A**) Clustering of a subset of Gmnn-associated genes that also exhibit Gmnn-dependent H3K9ac, by comparison with their expression levels in ES cells, embryonic CNS, and adult cortex. (**B**) Locations of Gmnn association or Gmnn-dependent H3K9ac are shown for several genes in (**A**) (WashU Epigenome Browser). (**C,D**) ES cells were transfected and selected to overexpress a Gmnn cDNA construct, and (**C**) qRTPCR and (**D**) immunoblotting demonstrate increased Gmnn expression levels at the mRNA and protein level. (**E**) Levels of expression of four Gmnn-associated genes were defined on days 1–3 of neural fate acquisition, with versus without Gmnn over-expression (Gmnn OE). Gene expression levels on each day of NE fate acquisition are expressed relative to ES = 1.0 and p-values shown (student’s t-test) compare expression with versus without Gmnn OE on each day of the NE fate acquistion: ** =< 0.01, * =< 0.05, ns = not significant. Error bars represent standard deviation for a representative qPCR performed in triplicate.

Our previous work suggested that Geminin over-expression promoted, while Geminin knockdown inhibited, histone acetylation at neural gene promoters^29^. Therefore, we defined profiles of histone H3 lysine 9 acetylation (H3K9ac) at gene promoters with and without Gmnn knockdown during NE fate acquisition, using an ES line that enables doxycycline-inducible knockdown of Gmnn^29^. These represent a subset of NE genes at which Gmnn levels control a transcriptionally active epigenetic status (Supplementary Table S4). One quarter of the Gmnn-associated genes in NE (24.5%) also exhibited Gmnn-dependent promoter acetylation (Fig. 3H). Genes that underwent Gmnn-dependent acetylation were associated with cell differentiation, development, and positive regulation of biological processes; the majority (64%) were more highly expressed in the embryonic CNS than in ES cells and this subset was enriched for development and neural development-related GO terms (Fig. 3I; Supplementary Fig. S5). Likewise, the subset of genes that are both Gmnn-associated in NE and undergo Gmnn-dependent histone acetylation exhibits predominantly enriched expression in embryonic CNS (73% of genes). This gene set contains transcription factors with later roles in neurogenesis (e.g. Nhlh1, Hes1, Dll3, Ebf2, Pax6, Vax1) and regional control of neural plate patterning or neuronal differentiation (e.g. Dlx1, Dbx1, Irx3, Lhx5, Pou4f1, Pax2, and Pax7) (Fig. 4A).

We focused on the criteria of Geminin association and regulation and CNS-enriched gene expression to select Gmnn-associated genes for further experimental work (Fig. 4A,B). Coincident with loss of pluripotency-associated gene expression and increased neural gene expression (Supplementary Fig. S6), expression levels of these genes increased during ES-NE fate acquisition *in vitro*. Gmnn overexpression during three days of ES-NE fate acquisition further increased expression levels of each gene tested (Fig. 4C–E). Interestingly, one of these genes was Zic1, which exhibits distinct peaks of Gmnn binding in ES cells and in NE (Fig. 4B). These data support a role for Gmnn in regulating the expression of embryonic CNS-expressed genes that are directly bound by Gmnn and whose epigenetic state (H3K9ac) is Gmnn-dependent. Integration of these features may select for key direct targets such as Zic1, through which Gmnn directly controls neural cell fate acquisition.

### Geminin and Zic1 co-associate with CNS-enriched genes controlling neural development

To better understand the role of Zic1 in regulating neural fate acquisition, we also performed ChIP-seq to define the Zic1 binding profile in ES-derived neuroectoderm and compared Geminin and Zic1 associated genes in NE. As described above, Gmnn associated with the Zic1 gene at distinct peak locations in ES cells and NE (Fig. 4B), while Gmnn over-expression could also promote Zic1 expression during neural fate acquisition (Fig. 4E), demonstrating a regulatory relationship between these neuroectodermal fate-promoting activities.

**Figure 5:**
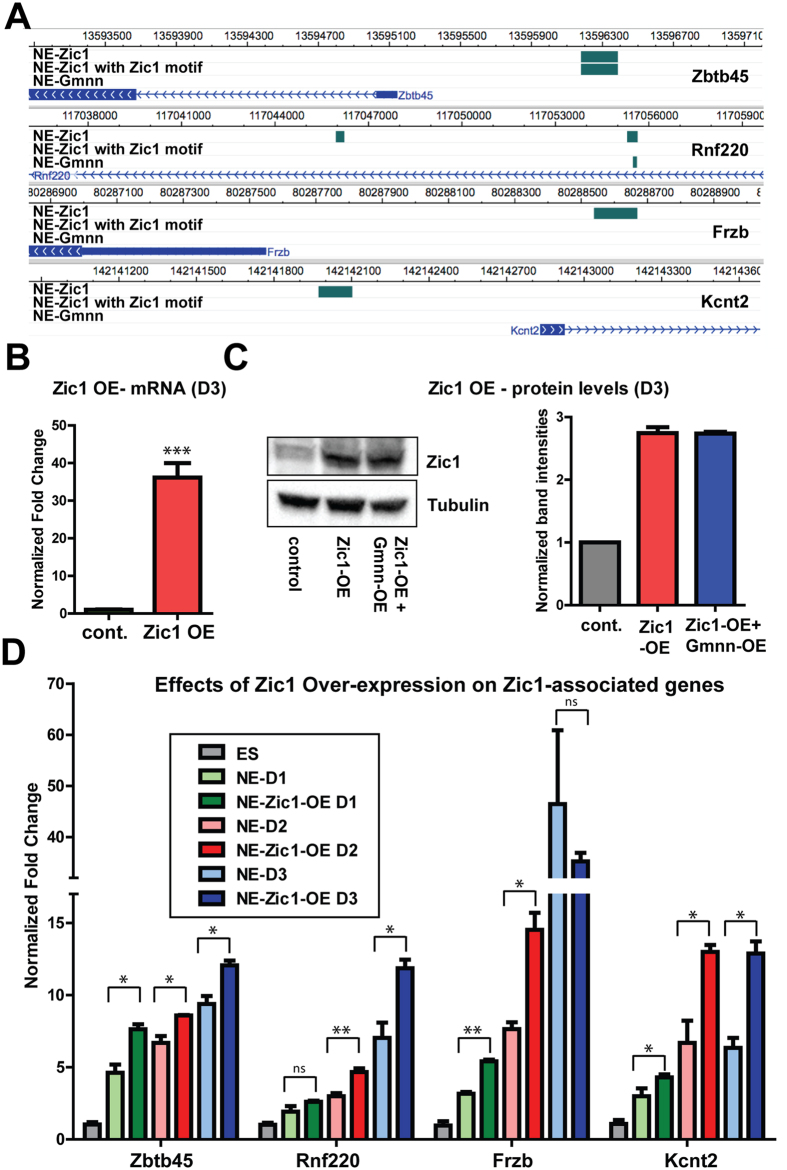
Zic1 over-expression during neural fate acquisition promotes the expression of Zic1-associated genes. (**A**) Browser views of selected Zic1-associated genes, tested below. (**B,C**) ES cells stably over-express Zic1 (Zic1 OE) after three days of neural cell fate acquisition (D3), as defined by qRTPCR for mRNA (**B**) and immunoblotting for protein (**C**). (**D**) Gene expression levels of Zic1-associated genes were defined by qRTPCR during 3 days of neural fate acquisition (D1-3), with versus without Zic1 overexpression. Gene expression levels on each day of NE fate acquisition are expressed relative to ES = 1.0 and p-values shown (student’s t-test) compare expression with versus without Zic1 OE on each day of the NE fate acquisition: ** =< 0.01, * =< 0.05, ns = not significant. Error bars represent standard deviation for a representative qPCR performed in triplicate.

We experimentally tested whether Zic1 over-expression could promote expression of a subset of genes that were predicted to be Zic1 associated in NE in our ChIP-seq data and that exhibited embryonic CNS-enriched expression (Fig. 5A). Expression of these genes increased during neural fate acquisition and Zic1 overexpression further promoted their expression during ES-NE fate acquisition (Fig. 5B–D). Therefore, comparison of Zic1-associated genes defined by ChIP-seq in NE with genes enriched in embryonic CNS *in vivo* can predict targets of Zic1-dependent activation during neural fate acquisition.

**Figure 6:**
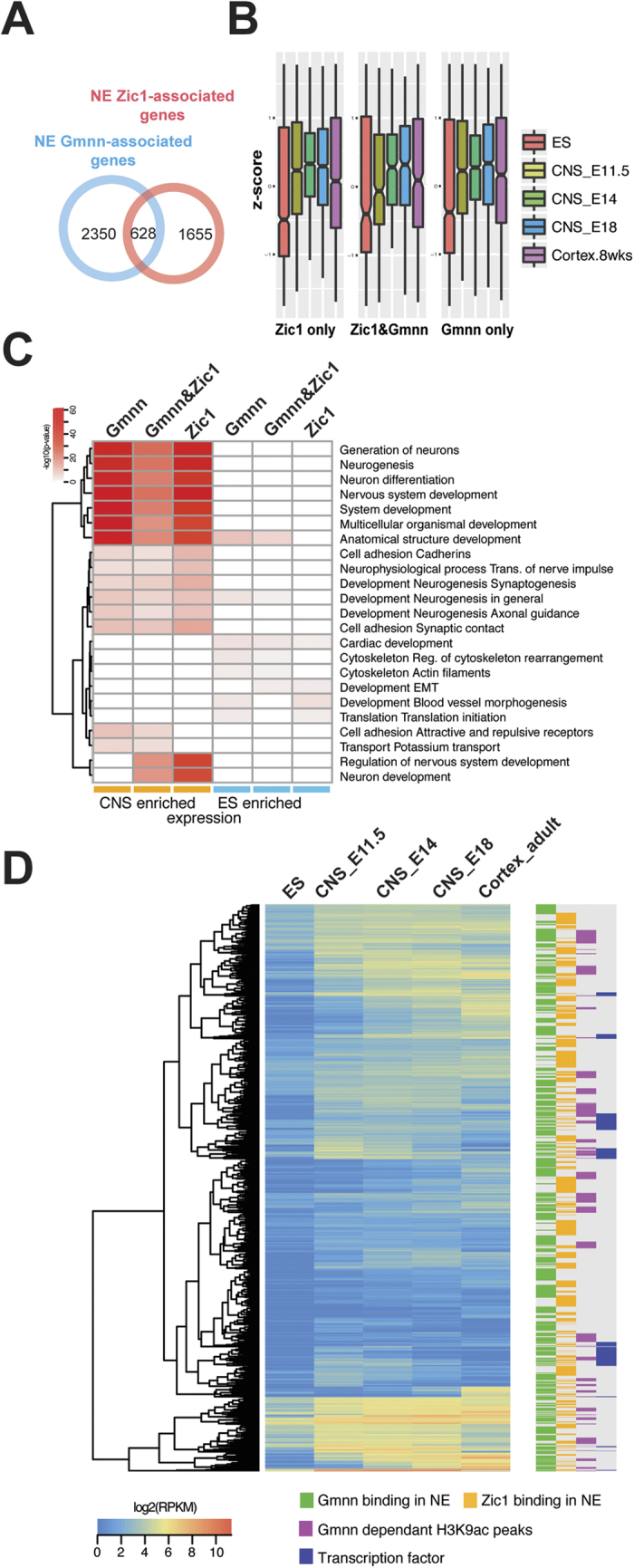
Comparison of Gmnn-associated and Zic1-associated genes in NE. (**A**) A subset of genes is associated with both Gmnn and Zic1. p-value (Chi-square test with Yates’ correction) <2.2 × 10^−16^. (**B**) z-scores for enrichment of expression in ES versus CNS tissues for all Zic and/or Gmnn associated genes. (**C**) GO enrichment analysis for associated genes with increased versus decreased expression in embryonic CNS, relative to ES cells. (**D**) Comparison of all Gmnn or Zic1 associated genes, subsets that undergo Gmnn-dependent acetylation, and transcription factors, with their relative enrichment of expression in embryonic CNS tissues.

We also compared Geminin- and Zic1-associated genes defined by ChIP-seq in NE. 3291 Zic1-associated peaks were defined; these corresponded to the nearest transcription start sites of 2283 genes, including 628 genes that also have Gmnn associated peaks in NE (Fig. 6A). Like the Gmnn-associated genes, the Zic1-associated gene set exhibited embryonic CNS-enriched expression (Fig. 6B). Gmnn and Zic1 association occurred through intersecting peak locations on the genome at some of these genes (201 intersecting peaks of Gmnn-Zic1 association), suggesting the potential for Gmnn and Zic1 cooperative activity in regulating their expression.

Many of the same GO terms were obtained for genes associated with Geminin only, Zic1 only, or both Geminin and Zic1 (Fig. 6C). Most Gmnn- and/or Zic1-associated genes exhibited embryonic CNS-enriched expression (Fig. 6D) and this gene set was enriched for terms related to neural development (neurogenesis, neuron differentiation) (Fig. 6C). By contrast, the smaller sets of Gmnn and/or Zic1-bound genes with higher expression in ES cells than in CNS were comprised of genes involved in alternate cell fate pathways and processes (e.g. cardiac or blood vessel development, cytoskeleton, EMT) (Fig. 6C).

### Enriched transcription factor consensus binding motifs at Geminin- and Zic1-associated peaks in neuroectoderm

**Figure 7:**
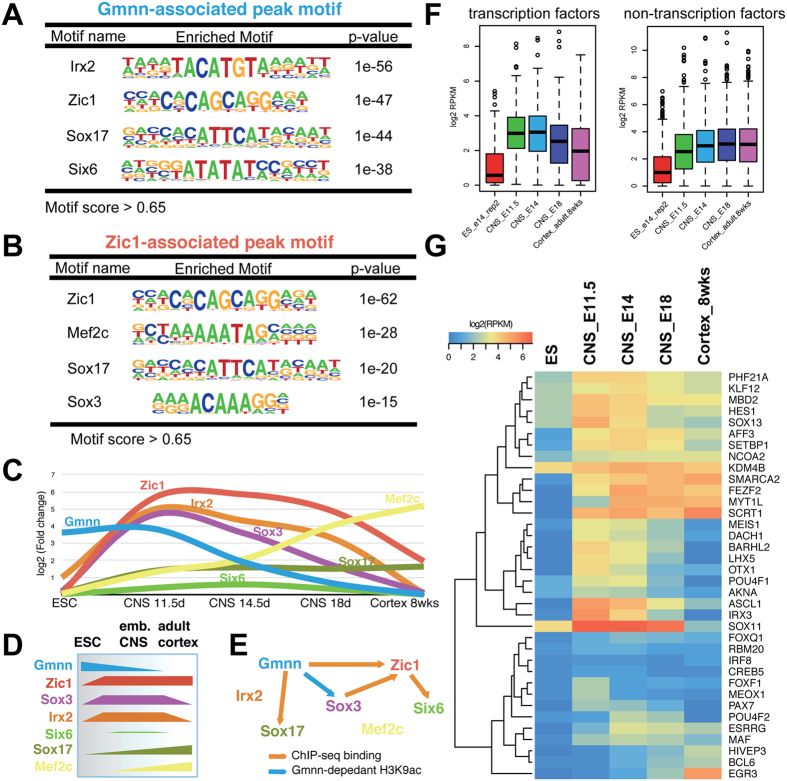
Enrichment of transcription factor consensus binding motifs in Gmnn- and Zic1-associated sequences in NE. (**A**) Gmnn-associated or (**B**) Zic1-associated peak sequences in NE were used to obtain the most highly enriched transcription factor consensus binding motifs. (**C**) Relative enrichment in ES cells and in embryonic-adult CNS was defined for Gmnn and Zic1, and for the transcription factors that associate with the consensus motifs in (**A,B**), with expression trends summarized in (**D**). (**E**) Associations between these transcription factors, as defined by these ChIP-seq data and Sox3 ChIP-seq analysis in ES-derived NE^35^. (**F,G**) Gmnn- and Zic1-associated transcription factors exhibit embryonic CNS enriched expression and include those with known roles in neural development.

To consider how Geminin and Zic1 may associate with the genome during neural fate acquisition, we examined sequence motifs enriched at their associated peaks in NE. Both Gmnn and Zic1 peaks in NE were enriched for Zic1 consensus binding sequence motifs, suggesting potential locations for Gmnn/Zic1 cooperative action in NE gene regulation (Fig. 7A,B). Gmnn-bound peaks in NE were also enriched for consensus binding motifs for two homeodomain transcription factors, Six6 and the Iroquois transcription factor Irx2 (Fig. 7A). Gmnn interactions have been described with homeodomain transcription factors, including several Hox transcription factors and Six3/Six6^15^,^17^,^31^. As both Irx2 and Six6 control regional neural plate patterning^32^,^33^,^34^, these or other homeodomain transcription factors that bind to these consensus motifs could potentially modulate Gmnn’s activity at these locations. Both Gmnn and Zic1-associated peaks also contain consensus motifs for Sox17, while Zic1 NE peaks were also enriched for consensus binding sequence motifs for Sox3 and Mef2c (Fig. 7A,B).

We assessed relative levels in ES cells and embryonic and adult CNS of transcription factors that recognize these consensus motifs, by comparison with Geminin and Zic1 (Fig. 7C,D). Sox3, Irx2, and Six6 are all expressed in the embryonic neural plate, with broad Sox3 expression from epiblast stages^24^, while Irx2 and Six3 exhibit regionally restricted neural expression^32^,^33^,^34^. Therefore, their expression is compatible with a potential role in gene co-regulation in embryonic NE, potentially acting through some of the same chromatin regions associated with Gmnn or Zic1 binding. In Xenopus early embryos, Zic1, Sox3, and Gmnn can regulate each other’s expression and all three activities contribute to establishing neural competence, neural fate acquisition, and maintaining the neural progenitor state. Our ChIP-seq data and that of others^35^,^36^ suggests that similar regulatory relationships may operate during mammalian neural fate acquisition, with Gmnn regulating Sox3 acetylation, while both Sox3 and Gmnn associate with the Zic1 gene during neural fate acquisition (Fig. 7E). Therefore, we further explored the potential of Gmnn and Zic1 to cooperatively control neural gene expression and defined the gene regulatory network under their control.

### Geminin and Zic1 cooperatively promote the expression of transcription factors that control neural development

**Figure 8:**
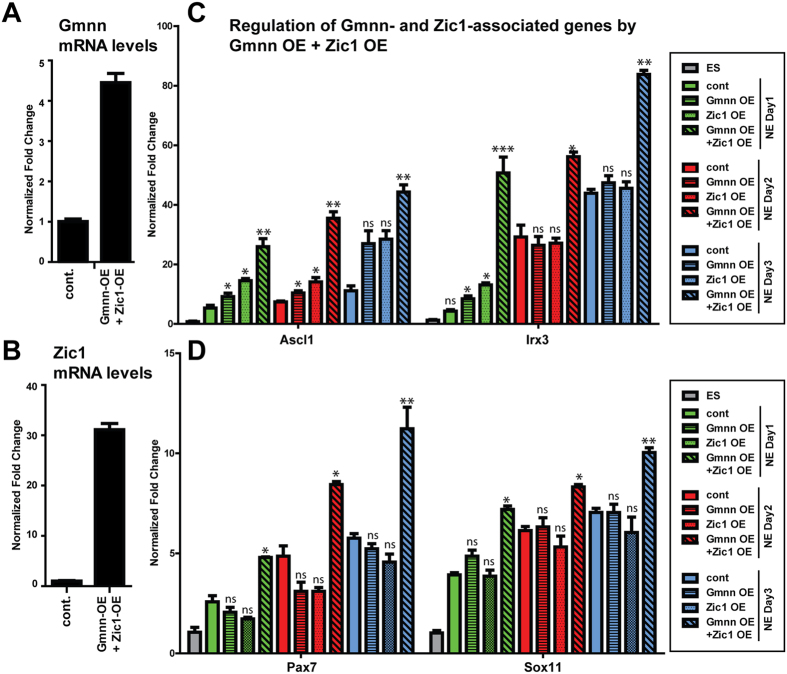
During neural cell fate acquisition, Gmnn and Zic1 cooperatively promote the expression of Gmnn- and Zic1-associated genes. ES cells that stably over-expressed both Gmnn and Zic1 were established and Gmnn (**A**) or Zic1 (**B**) mRNA levels were defined in over-expressing ES cells relative to non-overexpressing cells (cont.). Gmnn and Zic1 protein levels after combined Gmnn + Zic1 over-expression are shown in Figs 5D and 6C. (**C,D**) Expression levels of four Gmnn- and Zic1-associated genes were evaluated by qRTPCR on days 1–3 of neural fate acquisition, in response to single or combinatorial over-expression of Gmnn and/or Zic1. Gene expression levels on each day of NE fate acquisition are expressed relative to ES = 1.0 and p-values shown (student’s t-test) compare expression with versus without Gmnn and/or Zic1 OE at each day of NE fate acquisition: *** =< 0.001, ** =< 0.01, * =< 0.05, ns = not significant. Error bars represent standard deviation for a representative qPCR performed in triplicate.

In considering regulatory networks through which Geminin and Zic1 could control neural fate acquisition, associated genes encoding transcription factors with CNS-enriched expression may be of particular interest. Gmnn and Zic1 co-associated transcription factors exhibited CNS-enriched expression specifically at embryonic but not at adult stages (Fig. 7F), while many have known roles in regulating neural development (e.g. Ascl1, Sox11, Irx3, Pou4f1, Hes1, Pax7, Lhx5, Otx1, Myt1l)(Fig. 7G). Gmnn and Zic1 co-association with these genes could reflect a cooperative role in regulating their expression. To test this, we overexpressed either Gmnn or Zic1, or both Gmnn and Zic1 together, during neuroectodermal fate acquisition (Figs 4D, 5C and 8A,B) and assessed levels of four co-associated transcription factors (Fig. 7G) with roles in neurogenesis (Ascl1), regional neural plate patterning (Pax7, Irx3), or neuronal differentiation (Sox11). Neither Gmnn nor Zic1 over-expression singly was sufficient to elevate levels of these genes substantially during neuroectodermal fate acquisition, but combined over-expression of Gmnn and Zic1 cooperatively elevated expression levels of all four genes (Fig. 8C,D). Therefore, association of both Zic1 and Gmnn with subsets of genes encoding transcription factors that control neural development may represent one mechanism for promoting their expression.

## Discussion

In this study, we endeavored to understand the basis of neural fate acquisition by defining Geminin-associated genomic locations both in ES cells (where some regulators of neural competence, such as Geminin and Sox2, are already expressed) and in ES-derived neuroectoderm. We found that chromatin association of Gmnn changed during the ES-NE transition, but that most Gmnn-associated genes in both contexts exhibited embryonic CNS-enriched expression. These Gmnn-associated genes were preferentially enriched for GO terms associated with neural development, neurogenesis, and neuronal differentiation. Therefore, a major aspect of Gmnn’s role in regulating early cell fate acquisition is to associate with chromatin at neural genes and to promote expression of a gene program controlling neurodevelopment.

To define key Geminin targets that may account for its ability to promote neural fate, we intersected genes that are Geminin-associated in NE, undergo Geminin-dependent histone acetylation, and have embryonic CNS-enriched expression. Gmnn deficiency in a conditional mouse model causes neural tube defects through diminished expression of genes involved in neural specification, neurogenesis, neuronal differentiation and regional neural plate patterning (GSE42052)^28^. Interestingly, a subset of the Gmnn associated and regulated genes defined here in this *in vitro* model also exhibit diminished expression in the Gmnn-deficient E10.5 neural tube *in vivo*. Among Gmnn bound and regulated genes shown in Fig. 4A, Gmnn-dependent genes in the neural tube *in vivo* include transcription factors with roles in neurodevelopment (e.g. Bcl11a, Pou6f1, Lhx5, Pax2, Runx1t1, Pax7, Pou4f1, Tal1) and other genes that control neuronal development or function (e.g. Gabbr1, Lrrc4b, Rgs9, Nell1, Sema4a, Ecel1, Ephb1, Grin2d, Cadm4, Fgf11, Insm1, Rusc1). Gmnn’s direct association with and ability to promote the acetylation of these genes may represent an early event during neural cell fate acquisition both *in vivo* and *in vitro*, activating regulatory networks that promote neural fate acquisition and control later neural development.

Geminin does not contain a DNA binding domain. Therefore, an interesting question that our genome-wide data raises is how Gmnn associates with chromatin at neurodevelopmental genes to promote their expression. Gmnn interacts both with chromatin modifying complexes (SWI/SNF and Polycomb) and with homeodomain transcription factors, which could mediate Gmnn’s recruitment to specific chromatin locations in a temporal and cell type specific manner^15^,^16^,^17^,^31^. During neural fate acquisition of ES cells, Gmnn preferentially associates with acetylated chromatin and promotes an accessible and highly acetylated chromatin state at neural genes^29^.

Here, we defined the promoters subjected to this regulation throughout the genome by defining profiles of H3K9 acetylation with versus without Gmnn knockdown during neural cell fate acquisition. About one quarter of the Gmnn-associated genes in NE also underwent Gmnn-dependent histone acetylation. Most of these genes exhibit CNS-enriched expression and are involved in neural development.

In prior work, we tested mechanisms through which Geminin could influence chromatin acetylation and accessibility^29^. Gmnn enhanced chromatin accessibility and histone acetylation, both during neural fate acquisition of ES cells and in *in vitro* biochemical reconstitution experiments. In the latter work, recombinant Gmnn protein could enhance histone acetylation and chromatin accessibility rapidly in reconstituted *in vitro* assays. However, this required both use of a chromatin substrate and addition of ES nuclear extract to the reaction^29^. By constrast, Gmnn was unable to enhance histone acetylation in simpler reconstituted assays using histone peptide substrates and purified histone acetyltransferase (HAT) proteins^29^. Manipulation of Gmnn levels in ES cells also did not alter expression levels of HATs or histone deacetylases (HDACs) tested in this work^29^. These data suggest that Gmnn may promote histone acetylation indirectly, by influencing HAT occupancy or activity through effects on chromatin remodeling complexes that alter chromatin accessibility. Activation of gene transcription involves coordinated activities of chromatin regulatory activities that alter both chromatin accessibility and histone modification state, with some of these activities functionally interacting. For example, both SWI/SNF and Polycomb complexes can associate and cofunction with HATs and HDACs. The likely indirect nature of Gmnn’s effects on histone acetylation may account for findings that Gmnn-dependent changes in histone acetylation are only seen at about one quarter of Gmnn-associated genes and that Gmnn can promote the expression of some genes that did not exhibit Gmnn-dependent histone acetylation in our genome-wide studies.

The finding that Geminin associates with the Zic1 gene in both ES cells and in NE was of particular interest, as several findings suggest a functional relationship between Gmnn and Zic1 during neural cell fate acquisition. Similar to Gmnn’s ability to promote neural fate acquisition of ES cells, Zic1 is required for specification of ES cells as neuronal precursor cells in response to retinoic acid^37^. In mouse models, Zic1 and Zic3 are required together to regulate forebrain development and cooperate in promoting neural progenitor expansion, while inhibiting neuronal differentiation^38^, while Zic1 also promotes neural precursor formation and maintenance throughout the neural tube^39^. Gmnn likewise controls both the specification and maintenance of neural progenitor cells, while its expression is down-regulated during neuronal differentiation^29^,^40^,^41^. Therefore, both Gmnn and Zic1 have overlapping roles in promoting formation and maintenance of neuronal progenitors during early mammalian neural development.

Our work here suggests that these common Geminin and Zic1 activities may reflect direct association with and regulation of some shared target genes. We defined regulatory relationships between Gmnn and Zic1 during neural fate acquisition, as Gmnn associates with distinct peaks at the Zic1 gene in ES cells and in NE, while Gmnn over-expression promotes Zic1 expression. Both Zic1- and Gmnn-associated locations in NE were also enriched for Zic1 consensus binding sequence motifs, suggesting that Gmnn-Zic1 functional cooperation could regulate sets of genes involved in neural fate acquisition. While Zic1 targets in post-natal cerebellar development had been determined^42^,^43^, Zic1-associated locations during neural cell fate acquisition were unknown. Therefore, we conducted Zic1 ChIP-seq during neural fate acquisition in this study. This work defined a common set of target genes that associate with both Gmnn and Zic1, with a subset of these targets exhibiting co-association of Gmnn and Zic1 at the same peak location in neuroectoderm. One plausible mechanism for Gmnn recruitment to these locations would be by direct interaction with Zic1. We tested this by co-immunoprecipitation of epitope tagged versions of both proteins, but did not detect Gmnn-Zic1 interaction (Supplementary Figure S7). Therefore, Gmnn’s association with these genomic locations does not appear to be mediated by direct interaction with Zic1.

Association of Geminin and Zic1 at a common set of genes defines some transcriptional targets that may mediate their shared activities in neural progenitor specification and maintenance. We focused on the subset of Gmnn and Zic1 associated genes that exhibit CNS-enriched expression and encode transcriptional and epigenetic regulatory activities (Fig. 7F). These include transcription factors with central roles in regulation of neurogenesis (e.g. Ascl1), neural plate regionalization (Pax7/Irx3), and neuronal differentiation (Sox11). Interestingly, overexpression of Gmnn or Zic1 alone was insufficient to induce expression of these genes, but combined Gmnn and Zic1 overexpression cooperatively increased their expression during neural fate acquisition. Therefore, overlapping expression and activities of Gmnn and Zic1 in nascent neuroectoderm may reflect an additive or cooperative role for Gmnn and Zic1 in promoting the expression of transcription factors with key roles in later neural development.

In addition to Geminin and Zic1, several other transcriptional regulatory activities promote neural fate acquisition and neural progenitor maintenance. Notably, the SoxB1 family transcription factors Sox2 and Sox3, like Gmnn and Zic1, are broadly expressed in nascent neuroectoderm from gastrulation. These nTFs are also involved in neural progenitor formation and maintenance, while another SoxB1 TF, Sox11, acts to promote neuronal differentiation. Genome-wide characterization of their binding sites has defined sequential occupancy of some of the same genomic locations by Sox2, Sox3 and Sox11 in ES-derived neural progenitor cells^36^. Therefore, we compared genes associated with Zic1 and/or Gmnn with genes associated with each of these transcription factors in neural progenitor cells across studies, focusing on the subset of transcription factor-encoding genes with CNS-enriched expression (Fig. 7G). These analyses defined groups of Gmnn and Zic1-associated genes that are also associated with Sox2 and, in some cases, Sox3, in neural progenitors, while overlap with Sox11 was limited. We mapped regulatory relationships between these genes, as inferred from TF associations defined by ChIP-seq studies in ES-derived neural progenitor cells, to define a gene regulatory network (GRN) of Gmnn, Zic1, and/or Sox2/3-associated transcriptional regulatory activities with enriched expression in the embryonic CNS (Supplementary Fig. S8).

Common transcription factor targets associated with both Geminin and Zic1 in this GRN include: Ascl1, Sox11, Sox13, Irx3, Pax7, Otx1, Hes1, Lhx5, Pou4f1, and Barhl2 and we demonstrated that cooperative over-expression of Gmnn and Zic1 could increase expression levels of several of these transcription factors during neural fate acquisition, while over-expression of either Gmnn or Zic1 alone was not sufficient to induce their expression. Targets of Zic1, Gmnn, and Sox2 include Ascl1, Creb5, Otx1, Mbd2, Phf21a, Smarca2, Bcl6, Sox11, Scrt1 and Meis1, while genes associated with Sox2 and Gmnn also include other transcription factors such as Otx2, which acts in nascent neuroectoderm to promote neural and repress alternate mesodermal cell fate^44^. Interestingly, regulation of some of these genes could involve cooperativity between Zic1, Gmnn, and Sox2/3, since Gmnn and Zic1 targets overlap with those of Sox2 and Sox3, and Sox3 consensus binding motifs are enriched at Zic1-associated genomic regions.

This GRN incorporates relationships between transcription factors that control early acquisition of neural cell fate and those that regulate later aspects of neural development. Some genes are highly interconnected in the GRN, being associated with multiple nTFs and encoding transcription factors essential for later neural development. These may constitute central mediators of the early transcriptional response to establish neural cell fate. Their regulation by multiple nTFs during neural fate acquisition may link events occurring at the onset of neural cell fate acquisition to later neurogenesis, regional neural plate patterning, and neural progenitor maintenance and differentiation, coordinating and executing transcriptional programs that bridge the earliest and subsequent events of neural development.

## Methods

### Embryonic stem cell lines and CRISPR-mediated epitope tag knock-in

All mouse embryonic stem cell lines used are clonal derivatives of ES-E14TG2a (ATCC #CRL-1821). H3K9ac ChIP was performed in a clonal derivative of A2lox^45^; this was engineered for Doxycycline-inducible expression of a miR30-based shRNA and used previously to obtain effective Gmnn knockdown^29^. We also used CRISPR/Cas9-mediated genome engineering of ES-E14TG2a to generate clonal embryonic stem cell lines with knock-in of a Ty1 epitope tag sequence at the Gmnn or Zic1 gene. These lines express Ty1-tagged Gmnn or Zic1 proteins under the control of endogenous Gmnn or Zic1 regulatory sequences, and were used for ChIP-seq analysis. This allowed use of a ChIP grade Ty1 monoclonal antibody, as commercially available Gmnn and Zic1 polyclonal antibodies exhibited inconsistent performance, with lot-dependent variability, or were unsuitable for ChIP applications.

To generate clonal epitope tag knock-in lines, ES cells were transduced with a lentiviral vector for co-expression of the “nickase” form of Cas9 (Addgene pX335-U6-Chimeric_BB-Cbh-hSpCas9n(D18A) with addition of a hPGK-PuroR selection cassette) and guide RNA sequences directed against Gmnn (T CTC AGT ATG AAG CAG AAG CAG G) or Zic1 (GGA CCC CAG TAT CCC GCG ATT GG) and also with a donor plasmid to stimulate homology-directed repair after Cas9 cleavage. Donor plasmids contained either Gmnn or Zic1 sequence homology arms with a 3XTy1 tag and glycine linker sequence introduced in frame at the amino-terminus of Gmnn or Zic1. Silent mutations were introduced into these donor sequences (underlined) corresponding to the guide RNA cleavage sites in Gmnn or Zic1, to prevent Cas9-mediated cleavage of the donor plasmid sequences: Zic1: GGA CCC CAG TAT CCC GC**C** AT**C** GG) and Gmnn: T CTC AGT ATG AAG CAG AAG CA**A** G.

Genomic DNA and mRNA obtained from clonal ES lines were screened for the correct targeting event using primers to detect the Ty1-Zic1 junction in genomic DNA (Ty1 Forward: gac gcc gaa gtc cat aca aa and Reverse3-Zic1: gcg cct cga ggt cct aca tta att tcc ata cct g) or to detect the Ty1-Gmnn junction in mRNA (Ty1 forward and R-Gmnn: tct gga cca cag ctt gaa gt) and expression of Ty1 epitope tagged Gmnn and Zic1 was detected in ES cells and ES-derived neuroectoderm by western blotting and immunocytochemistry.

### ES cell growth and directed differentiation

ES lines were routinely propagated on feeder cells (mouse embryonic fibroblast cells, Chemicon) in DMEM (Invitrogen) supplemented with 15% fetal calf serum (Hyclone), 1 mM sodium pyruvate, 1 mM Non-Essential amino acids, 1mM L-Glutamine (Invitrogen), 10^−4^ M 2-mercaptoethanol (Sigma) and 10^3^ units per ml of leukemia inhibitory factor (ESGRO, Chemicon). Monolayer neural differentiation of ES cells was done as previously described^6^. Briefly, ES cells cultured on feeder cells were dissociated and plated at 1 × 10^5^/cm^2^ for one day on 0.1% gelatin-coated tissue culture dishes. After 24 hours, cells were plated onto 0.1% gelatin-coated tissue culture dishes at low density (1 × 10^4^ cells/cm^2^) in N2B27 medium and cultured for 3 days.

### ES transfection and selection

Full length murine Geminin (MR202178) and Zic1(MR207138) cDNAs in expression vectors were purchased from Origene. For transfection and selection, feeder subtracted ES cells were transfected with 5 μg of DNA using Lipofectamine 2000 (Invitrogen) following the manufacturer’s protocol. 6 hrs after transfection the medium was changed to fresh ES medium and 24 hours after transfection the cells were replated on gelatin in ES media with LIF. 24 hrs after replating the cells were selected for stable transfectants using 300 μg/ml G418 for 6 days.

### qRT-PCR and immunoblotting

Total RNA was extracted using Clontech Nucleospin RNA extraction kit (#740955.250) using the manufacturer’s protocol. cDNA was synthesized using the Bio Rad iScript Reverse Transcriptase Supermix (#170-8841) followed by quantitative RT-PCR analysis using the Fast SYBR green mix from Applied Biosystems (#4385612) and gene-specific primer sequences shown in Supplementary Table S5. Anti-Gmnn antibody from Santa Cruz (Cat. No. #sc-13015) and anti-Zic1 antibody from Rockland Inc. (Cat. #200-401-159) were used for immunoblotting at 1:200 dilution. The Gmnn and Zic1 bands were quantified using Bio-Rad Image Lab software. Co-immunoprecipitation experiments were performed as previously described^16^ in HEK 293 cells transfected with HA-tagged Gmnn and Myc-tagged Zic1. Immunoprecipitation was performed with the HA tag using HA antibody (Abcam: ab9110) and blotted for Myc using Myc antibody (Abcam: ab32).

### Quantitative Chromatin Immunoprecipitation

Quantitative ChIP was done with modifications to a standard protocol (Upstate/Millipore) as follows. For each ChIP reaction, sheared chromatin (sonicated to 200–500 bp in 50 mM Tris-HCl, pH8.1, 10 mM EDTA, plus protease inhibitors) from 5 × 10^6^ ES cells was fixed in 1% Formaldehyde (Sigma) in DMEM +5% FBS and incubated with 5 μg of each antibody and Protein G Dynabeads (Invitrogen) as per the manufacturers instructions. For Ty1 ChIP, 1% SDS was also included in the shearing buffer. Antibodies used for ChIP were Gmnn (N-18; Santa Cruz sc-8449), H3K9ac (Abcam; Ab10812), and Ty1 (Diagenode C15200054). After washing (0.1% SDS, 1% TritonX, 2 mM EDTA, 20 mM Tris pH8.0, 150 mM NaCl), elution and cross-link reversal, DNA from each ChIP sample and the corresponding input sample was purified and analyzed further using qPCR as follows: each ChIP sample and a range of dilutions of the corresponding input sample (0.01–5% input) were quantitatively analyzed with gene specific primers using the 7500 FAST Real-time PCR Detection System (ABI) and SYBR Advantage qPCR Premix (Clontech). ChIP qPCR primers are in Supplementary Table S5.

### Genome-wide location analysis

For ChIP-seq sample preparation, two independent biological replicates per clone and two clones for each sample type were sequenced after ChIP for Ty1-Gmnn in ES cells and for Ty1-Gmnn and Zic1 in ES-derived neuroectoderm after 3 days of N2B27 monolayer culture. Control samples sequenced for both ES cells and ES-derived neuroectoderm included input and Ty1 ChIP performed in the E14TG2a ES line that does not express a Ty1-tagged fusion protein; subtraction of these controls after sequencing was used to define specific peaks of Gmnn and Zic1 ChIP enrichment. Samples underwent linear amplification using the SeqPlex whole genome amplification kit (Sigma). Illumina library preparation for sequencing was performed by the Genome Technology Access Center at Washington University School of Medicine and sequencing was performed on the HiSeq 2500 (single end reads).

ChIP-chip hybridization to tiled promoter arrays was performed on three independent biological replicates for the following sample types: Gmnn ChIP in ES cells and ES-derived neuroectoderm after 3 days of N2B27 monolayer culture, using the Gmnn N18 antibody (Santa Cruz sc-8449), and H3K9ac (Abcam; Ab10812 antibody) ChIP in ES-derived neuroectoderm with or without inducible Gmnn knockdown by addition of 500ng Dox to ES cells engineered for Dox-inducible Gmnn knockdown^29^. ChIP samples and corresponding input chromatin samples were hybridized to HD2 2.1 M feature Nimblegen tiled promoter arrays after processing ChIP samples with the Sigma WGA kit (#WGA2). Analysis with Nimblescan/Signal Map platform software defined scaled log2-ratio peak files, and Gmnn peaks with scores of fdr <0.05 and H3K9ac peaks with scores of fdr <0.2 that were present in at least two of three replicates were retained. For H3K9ac, peaks present in at least two of the three independent biological replicates after Dox-induced Gmnn knockdown were subtracted from those present in the control without Gmnn knockdown (no Dox) to define H3K9ac peaks that were sensitive to Gmnn deficiency.

In our present study, high stringency conditions were used for ChIP-seq with the Ty1-tagged Gmnn and Zic1 proteins, since Ty1 is not endogenously expressed in ES cells (see Methods). This resulted in relatively fewer peaks for both data sets, in comparisons with other published ChIP-seq datasets. Furthermore, there was limited overlap with chromatin-associated peaks obtained for Gmnn using the ChIP-chip and ChIP-seq approaches. This may reflect the lower levels of Ty1-Gmnn expression relative to endogenous Gmnn (Fig. S2) or more stringent ChIP conditions (see Methods). Therefore, we combined peaks obtained from both approaches and performed cross-validation of a subset of Gmnn peaks by ChIP qPCR, using both the N18 Gmnn antibody (used for ChIP-chip) and the Ty1 antibody in the tagged line (used for ChIP-seq). We obtained significant enrichment of ChIP-chip and ChIP-seq peaks with both validation methods. Hence, this approach yielded high confidence chromatin binding events for Gmnn.

### RNA-seq data analysis

Raw read (SRA) of RNA-seq data was downloaded from the mouse ENCODE project^46^ as GEO accession GSE49847. Raw reads were aligned to the mouse genome (assembly mm9) using STAR version 2.4.2a^47^. Gene counts were derived from the number of uniquely aligned unambiguous reads by Subread:featureCount^48^, version 1.4.6, with GENCODE gene annotation (M1)^49^. Expression levels of genes in each sample were normalized to Reads Per Kilobase of transcript per Million mapped reads (RPKM), and z-score of genes were calculated as:


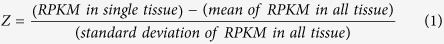


### Motif analysis

Motif enrichment analysis in Zic1 and Gmnn binding peaks was performed using the HOMER tool^50^. HOMER scanned the sequences of dsDMRs for known motifs, including the HOMER-provided motif library and JASPAR^51^ core vertebrate motifs, and calculated enrichment score P-values using hypergeometric testing.

### Enrichment for genomic features

CpG islands and refGene features (including 5′ UTR, exons, introns, and 3′ UTRs) were all downloaded from the UCSC Genome Browser^52^. Promoters were defined as 2.5 kb around the most 5′ transcription start site (2 kb upstream of and 0.5 kb downstream from TSS) of any refGene record. Intergenic regions were defined as regions between neighboring refGene loci. Fantom enhancer annotation was downloaded from FANTOM (http://fantom.gsc.riken.jp/data/)^53^. The enrichment of each genomic feature was calculated as:


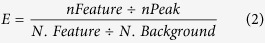


where nFeature is the number of ChIP peaks that contain genomic annotation features; nPeak is the total identified ChIP peaks; N.Feature is the number of each genomic annotation feature; N.Background is the number of windows in the mouse genome (mm9), which is calculated based on averaged length of each feature (CpG island: 601 bp, Promoter: 2500 bp, enhancer: 271 bp).

## Additional Information

**Accession codes:** ChIP-seq and ChIP-chip data sets were deposited into GEO as GSE81595 (Superseries of datasets GSE77246 and GSE81450).

**How to cite this article**: Sankar, S. *et al*. Gene regulatory networks in neural cell fate acquisition from genome-wide chromatin association of Geminin and Zic1. *Sci. Rep.* **6**, 37412; doi: 10.1038/srep37412 (2016).

**Publisher's note:** Springer Nature remains neutral with regard to jurisdictional claims in published maps and institutional affiliations.

## Supplementary Material

Supplementary Information

Supplementary Tables S1–S5
