# Supplementary material for: Gene regulatory networks in neural cell fate acquisition from genome-wide chromatin association of Geminin and Zic1
Source: Sci Rep. 2016 Nov 24;6:37412. doi: 10.1038/srep37412 (PMC5121602; doi:10.1038/srep37412)
Supplement: Supplementary Information [file srep37412-s1.pdf]

Supplementary Material

Gene regulatory networks in neural cell fate acquisition from genome-wide chromatin association of Geminin and Zic1. Savita Sankar, Dhananjay Yellajoshyula, Bo Zhang, Bryan Teets, Nicole Rockweiler, and Kristen L. Kroll

Fig. S1. Relative transcript expression levels in tissue-specific RNA-seq data sets. Range of expression for all RNA transcripts in each tissue-specific RNA-seq data set used for comparisons is expressed as a z-score (see Methods).

Fig. S2. Embryonic stem cell lines with knock-in of a 3XTy1 epitope tag at the Gmnn or Zic1 locus. (A) Ty1-Gmnn mRNA detected in clones 3 and 66, using Ty1 forward and Gmnn mRNA reverse primers. (B) Amplification across the Ty1-Zic1 junction in genomic DNA from Ty1-Zic1 clones 6 and 8 and subclones (using Ty1 forward-R3 Zic1 primers). (C) Expression of Ty1-Gmnn detected in targeted ES cells by immunoblotting for Ty1 (top panel, green) and Gmnn (bottom panel, N18 antibody), with GAPDH (red) as a loading control. NT control=non-targeted ES-E14TG2a ES cell line.

Fig. S3. ChIP-seq dataset read data. ChIP-seq data sets were obtained for two biological replicates per clone (aggregated reads shown) and for two clones per sample type, after performing Gmnn ChIP-seq in ES cells and in NE and Zic1 ChIP-seq in NE. Control samples (cont ES/NE) involved Ty1 ChIP performed in parallel using the same ES cell line (ES-

E14TG2a) that does not express the Ty1-Gmnn or Ty1-Zic1 proteins. Total, mapped, and non-redundant uniquely mapped reads are shown.

Fig. S4. ChIP qPCR validation of ChIP-seq and ChIP-chip peaks in ESCs. (A) Examples of peaks obtained for Gmnn by ChIP-seq in both ES cells and in NE. (B) ChIP qPCR with the Ty1 antibody was performed in the Ty1-Gmnn knock-in ES line and in ES-derived NE. (C-D) ChIP-qPCR validation of peaks obtained for Gmnn using ChIP-chip in ES cells, using either the N18 Gmnn antibody used for ChIP-chip (C) or using the Ty1 antibody in the Ty1-Gmnn knock-in ES line. In A, B and D, control samples (cont.) involved performing Ty1 ChIP in parallel with experimental samples using an ES line that does not express Ty1-tagged Gmnn. Negative=mouse negative control ChIP primer set1 from Active Motif (see **Supplementary Table S5**). p-values: ***=<0.001 and **=<0.01. Error bars represent standard deviation of a representative qPCR performed in triplicate.

Fig. S5. GO analysis of genes that are Gmnn-associated in ES cells or in NE or that undergo Gmnn-dependent H3K9 acetylation. Results are shown for all genes (left) or for those genes with two-fold enriched expression in ES cells relative to E14 CNS (right).

Fig. S6. Gmnn and Zic1 expression during ES-NE fate acquisition. Expression levels of pluripotency and neural marker genes and of Gmnn and Zic1 were defined in ES cells and in ES cell-derived NE at days 1-3 by quantitative RTPCR. Fold changes are shown relative to levels in ES cells (=1.0). Error bars represent standard deviation for a representative qPCR performed in triplicate.

Fig. S7. Geminin and Zic1 do not co-immunoprecipitate. HA-tagged Gmnn and Myc-tagged Zic1 were co-transfected into HEK293 cells and nuclear extracts were immunoprecipitated (IP) with an HA antibody. While both proteins were expressed, as detected by HA and Myc immunoblotting (IB), there was no evidence of Gmnn-Zic1 interaction.

Fig. S8. Gene regulatory networks in neural cell fate acquisition. Gmnn- and Zic1-associated genes in NE that encode transcription factors and epigenetic regulatory activities were assessed for co-association with Sox2 and Sox3 in ES-derived NE (see text). The set of these genes that exhibit CNS-enriched expression (>two-fold greater expression in E14 CNS, relative to ES cells), and were associated with Gmnn and/or Zic1 plus Sox2 and/or Sox3 were used to build gene regulatory networks showing associations.

Tables S1-S4. Gmnn-associated peak locations in ES-derived neuroectoderm (NE)(Table S1) and in ES cells (Table S2), Zic1-associated peaks in NE (Table S3), and promoters subjected to Gmnn-dependent H3K9 acetylation (Table S4). Peak locations and nearest transcription start site are indicated. Complete sets of raw and processed data, from which these final peak lists were derived, were deposited in GEO as GSE81595 (Superseries of datasets GSE77246 and GSE81450).

Table S5. Primers used for ChIP qPCR and RTPCR analysis. (A-B) ChIP qPCR primer pairs for (A) Gmnn-associated locations (B) and Zic1-associated locations. (C) qRTPCR primer pairs.

Figure S1

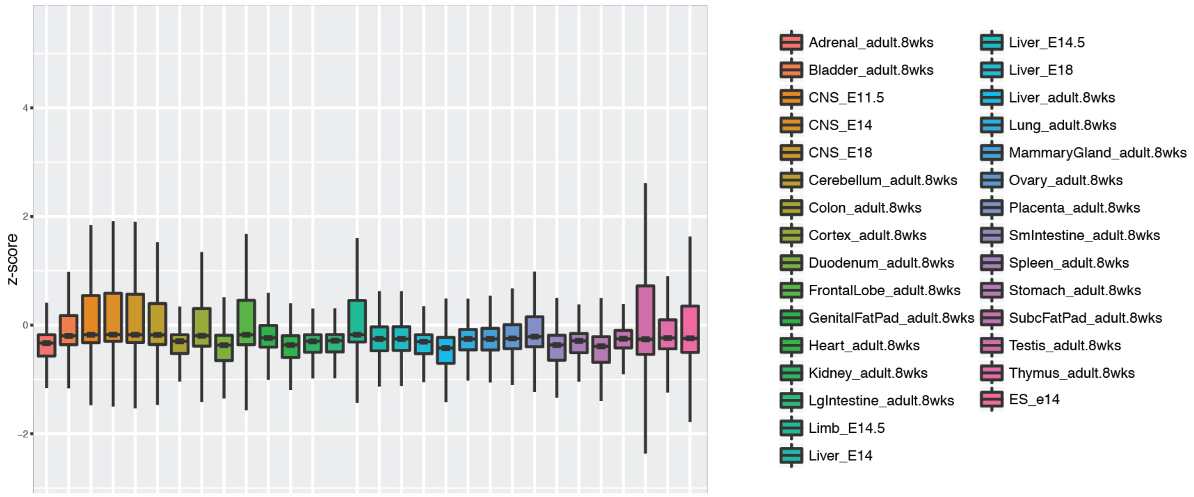

z-score
4
2
0
-2
Adrenal_adult.8wks
Bladder_adult.8wks
CNS_E11.5
CNS_E14
CNS_E18
Cerebellum_adult.8wks
Colon_adult.8wks
Cortex_adult.8wks
Duodenum_adult.8wks
FrontalLobe_adult.8wks
GenitalFatPad_adult.8wks
Heart_adult.8wks
Kidney_adult.8wks
LgIntestine_adult.8wks
Limb_E14.5
Liver_E14
Liver_E14.5
Liver_E18
Liver_adult.8wks
Lung_adult.8wks
MammaryGland_adult.8wks
Ovary_adult.8wks
Placenta_adult.8wks
SmIntestine_adult.8wks
Spleen_adult.8wks
Stomach_adult.8wks
SubcFatPad_adult.8wks
Testis_adult.8wks
Thymus_adult.8wks
ES_e14

Figure S2

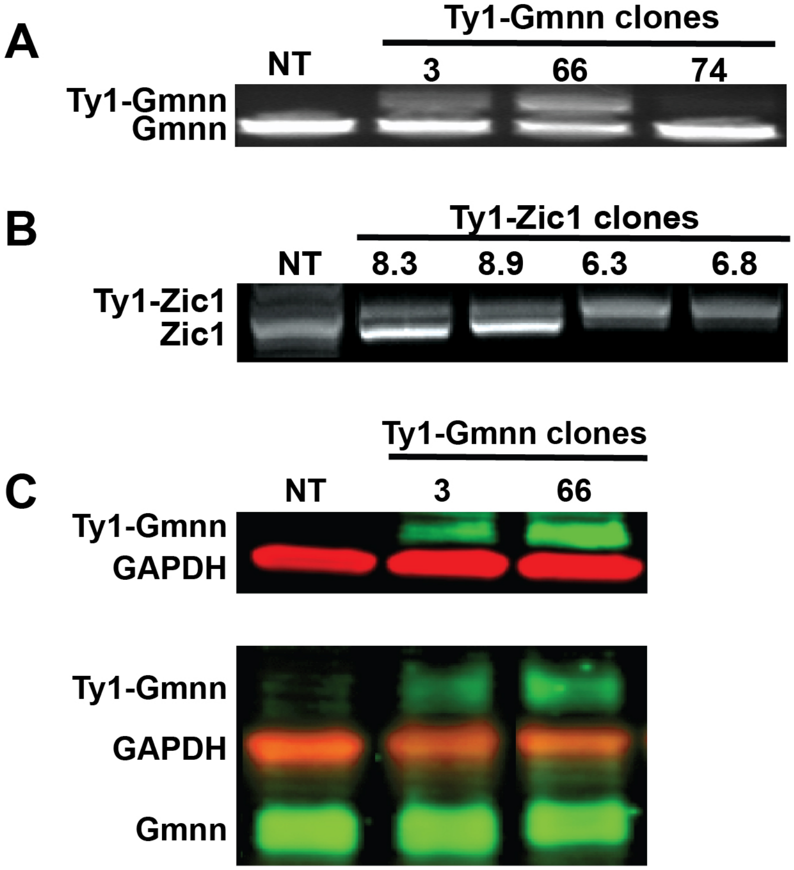
A
Ty1-Gmnn clones
NT
3
66
74
Ty1-Gmnn
Gmnn
B
Ty1-Zic1 clones
NT
8.3
8.9
6.3
6.8
Ty1-Zic1
Zic1
C
Ty1-Gmnn clones
NT
3
66
Ty1-Gmnn
GAPDH
Ty1-Gmnn
GAPDH
Gmnn

Figure S3

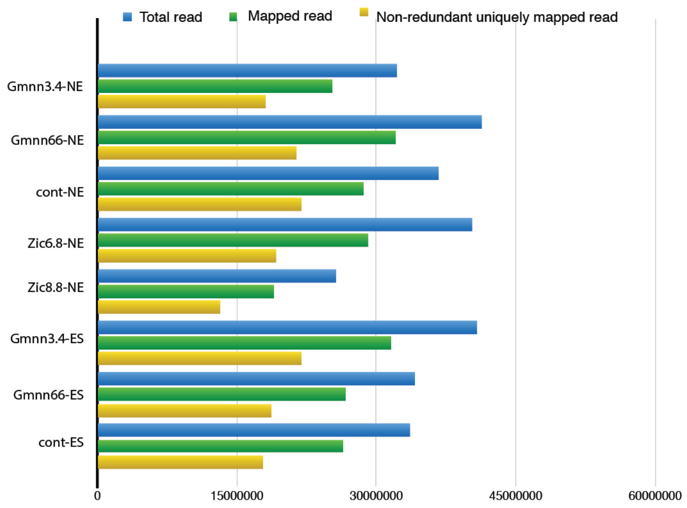

	Gmnn3.4-NE	Gmnn66-NE	cont-NE	Zic6.8-NE	Zic8.9-NE	Gmnn3.4-ES	Gmnn66-ES	cont-ES	Input
total reads	32271389	41429992	36816796	40445252	25701844	40989947	34222784	33725186	25989692
mappable reads	25413656	32208963	28710837	29234245	19021306	31728070	26843369	26476682	21599940
uniquely mapped reads	25413656	32208963	28710837	29234245	19021306	31728070	26843369	26476682	21599940
non-redundant uniquely mapped reads	18153580	21495096	21974552	19345193	13263067	21999569	18859857	17897415	14617909

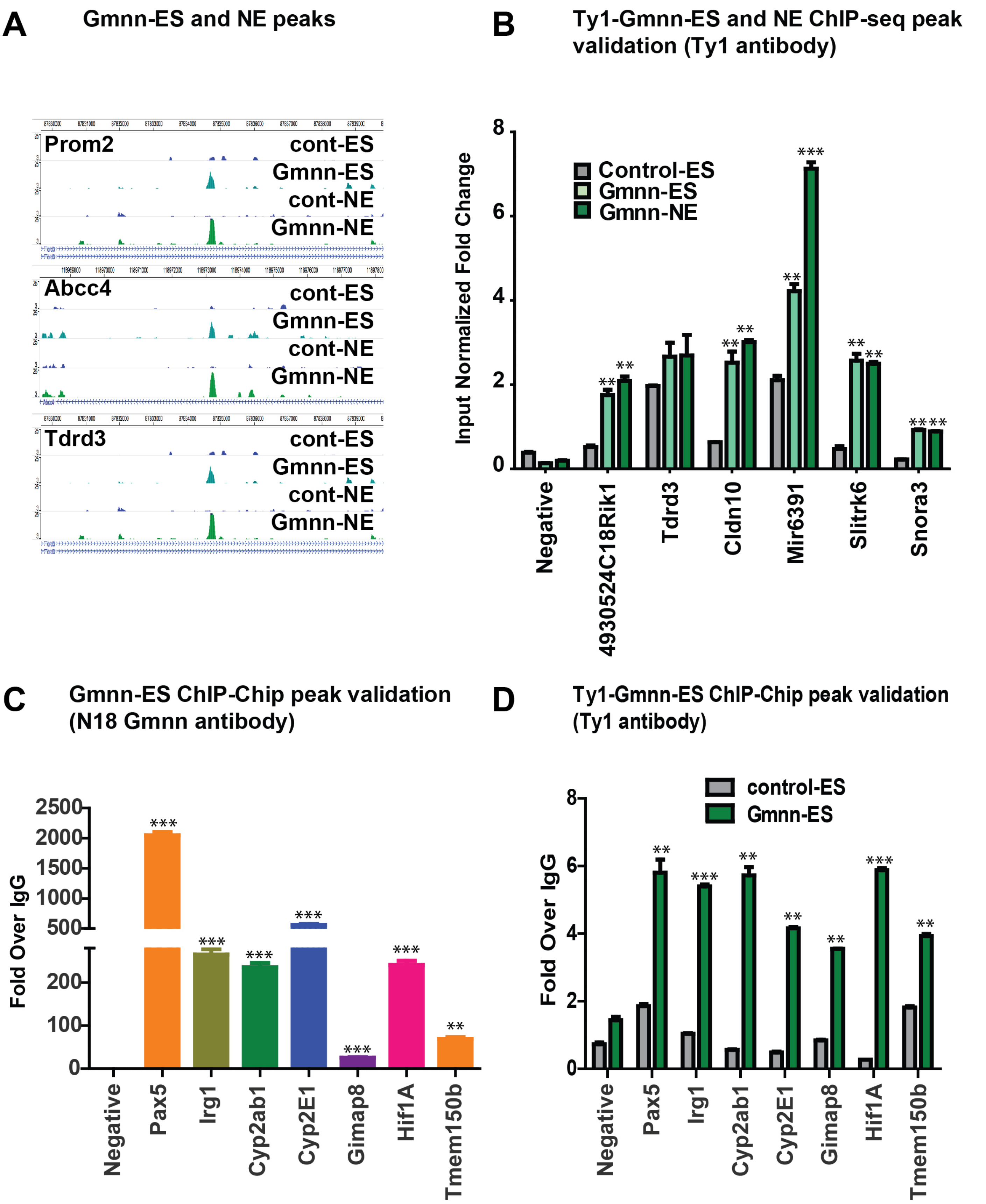
A
Gmnn-ES and NE peaks
Prom2
cont-ES
Gmnn-ES
cont-NE
Gmnn-NE
Abcc4
cont-ES
Gmnn-ES
cont-NE
Gmnn-NE
Tdrd3
cont-ES
Gmnn-ES
cont-NE
Gmnn-NE
B
Ty1-Gmnn-ES and NE ChIP-seq peak validation (Ty1 antibody)
Input Normalized Fold Change
Control-ES
Gmnn-ES
Gmnn-NE
Negative
4930524C18Rik1
Tdrd3
Cldn10
Mir6391
Slitrk6
Snora3
C
Gmnn-ES ChIP-Chip peak validation (N18 Gmnn antibody)
Fold Over IgG
Negative
Pax5
Irg1
Cyp2ab1
Cyp2E1
Gimap8
Hif1A
Tmem150b
D
Ty1-Gmnn-ES ChIP-Chip peak validation (Ty1 antibody)
control-ES
Gmnn-ES
Fold Over IgG
Negative
Pax5
Irg1
Cyp2ab1
Cyp2E1
Gimap8
Hif1A
Tmem150b

Figure S5

GmnnES associated genes, all

#	GmnnES bound-Processes	pValue
1	neurogenesis	1.95E-10
2	cell differentiation	3.42E-10
3	regulation of neurological system process	4.43E-10
4	generation of neurons	1.29E-09
5	nervous system development	1.37E-09
6	cellular developmental process	1.93E-09
7	regulation of localization	1.34E-08
8	system development	1.77E-08
9	regulation of multicellular organismal process	1.78E-08
10	neuron differentiation	3.05E-08
#	GmnnES bound-Networks	pValue
1	Muscle contraction	1.36E-03
2	Development_Neurogenesis in general	1.69E-03
3	Protein folding_Protein folding nucleus	3.38E-03
4	Signal Transduction_TGF-beta, GDF and Activin signaling	7.04E-03
5	Transport_Synaptic vesicle exocytosis	9.12E-03
6	Reproduction_Feeding and Neurohormone signaling	1.03E-02
7	Transport_Iron transport	1.88E-02
8	Cardiac development_Wnt_beta-catenin, Notch, VEGF, IP3 and integrin signaling	3.01E-02
9	Chemotaxis	3.61E-02
10	Development_Blood vessel morphogenesis	3.99E-02

GmnnES associated genes, ES enriched exp.

#	GmnnES-down ES-CNS-Processes	pValue
1	protein glucuronidation	2.17E-08
2	flavone metabolic process	1.50E-07
3	coumarin catabolic process	2.98E-07
4	coumarin metabolic process	3.71E-07
5	phenylpropanoid metabolic process	4.91E-07
6	regulation of glucuronosyltransferase activity	5.33E-07
7	negative regulation of glucuronosyltransferase activity	5.33E-07
8	negative regulation of cellular glucuronidation	5.33E-07
9	phenylpropanoid catabolic process	5.33E-07
10	amino acid import into cell	8.83E-07
#	GmnnES-down ES-CNS-Networks	pValue
1	Cell adhesion_Leucocyte chemotaxis	4.01E-04
2	Signal Transduction_TGF-beta, GDF and Activin signaling	2.25E-03
3	Cell adhesion_Cell junctions	2.90E-03
4	Reproduction_Feeding and Neurohormone signaling	1.04E-02
5	Cell adhesion_Glycoconjugates	1.38E-02
6	Signal Transduction_Cholecystokinin signaling	1.40E-02
7	Inflammation_NK cell cytotoxicity	1.45E-02
8	Inflammation_Protein C signaling	1.49E-02
9	Cytoskeleton_Actin filaments	1.91E-02
10	Cell cycle_Mitosis	2.04E-02

Gmnn NE associated genes, all

#	GmnnNE-Processes	pValue
1	multicellular organismal development	1.30E-57
2	system development	4.59E-57
3	single-organism developmental process	2.28E-51
4	developmental process	4.57E-51
5	anatomical structure development	5.40E-50
6	nervous system development	5.55E-44
7	organ development	2.01E-41
8	cell differentiation	9.94E-39
9	anatomical structure morphogenesis	2.94E-37
10	cellular developmental process	3.15E-37
#	GmnnNE-Networks	pValue
1	Development_Neurogenesis in general	1.37E-08
2	Development_Hedgehog signaling	8.00E-07
3	Cardiac development_Wnt_beta-catenin, Notch, VEGF, IP3 and integrin signaling	1.91E-06
4	Cell adhesion_Cadherins	5.36E-06
5	Signal transduction_WNT signaling	8.09E-05
6	Cardiac development_BMP_TGF_beta_signaling	8.58E-05
7	Cell adhesion_Synaptic contact	1.05E-04
8	Cell adhesion_Attractive and repulsive receptors	1.28E-04
9	Development_Neurogenesis_Axonal guidance	1.63E-04
10	Signal transduction_NOTCH signaling	3.27E-04

Gmnn NE associated genes, ES enriched exp.

#	GmnnNE-down ES-CNS-Processes	pValue
1	tissue development	1.15E-09
2	multicellular organismal development	1.44E-09
3	single organism reproductive process	1.82E-09
4	single-organism developmental process	2.00E-09
5	developmental process	3.99E-09
6	cell differentiation	6.38E-09
7	system development	7.87E-09
8	anatomical structure development	1.16E-08
9	G-protein coupled receptor signaling pathway coupled to cGMP nucleotide second messenger	1.67E-08
10	reproductive process	1.73E-08
#	GmnnNE-down ES-CNS-Networks	pValue
1	Cardiac development_Wnt_beta-catenin, Notch, VEGF, IP3 and integrin signaling	7.11E-04
2	Development_Neurogenesis in general	2.02E-03
3	Development_Blood vessel morphogenesis	3.39E-03
4	Cytoskeleton_Regulation of cytoskeleton rearrangement	3.87E-03
5	Development_Skeletal muscle development	5.48E-03
6	Cell adhesion_Amyloid proteins	1.64E-02
7	Translation_Translation initiation	1.72E-02
8	Cytoskeleton_Actin filaments	2.06E-02
9	Cytoskeleton_Intermediate filaments	2.19E-02
10	Immune response_Phagocytosis	3.78E-02

Gmnn-dep H3K9ac associated genes, all

#	Gmnn-dep H3K9ac-Processes	pValue
1	positive regulation of biological process	1.60E-50
2	positive regulation of cellular process	2.05E-48
3	cellular component organization or biogenesis	8.11E-47
4	cellular component organization	1.45E-46
5	cellular metabolic process	7.46E-46
6	positive regulation of metabolic process	3.42E-41
7	metabolic process	2.18E-39
8	organic substance metabolic process	2.64E-38
9	negative regulation of biological process	1.65E-37
10	negative regulation of cellular process	2.95E-37
#	Gmnn-dep H3K9ac-Networks	pValue
1	Signal transduction_ESR2 pathway	2.28E-09
2	Development_Hemopoiesis, Erythropoietin pathway	1.18E-07
3	Cell cycle_G1-S Interleukin regulation	1.75E-07
4	Cell cycle_G1-S Growth factor regulation	1.89E-07
5	Development_Neurogenesis_Axonal guidance	1.26E-06
6	Signal transduction_ESR1-nuclear pathway	4.73E-06
7	Development_Blood vessel morphogenesis	6.62E-06
8	Cytoskeleton_Regulation of cytoskeleton rearrangement	1.18E-05
9	Signal transduction_WNT signaling	1.38E-05
10	Development_Regulation of angiogenesis	1.83E-05

Gmnn-dep H3K9ac associated genes, ES-enriched exp.

#	Gmnn-dep H3K9ac-down ES-CNS-Processes	pValue
1	negative regulation of biological process	2.34E-15
2	negative regulation of cellular process	1.03E-14
3	response to organic substance	8.16E-14
4	negative regulation of biosynthetic process	1.27E-12
5	regulation of vasodilation	1.42E-12
6	protein targeting to membrane	1.75E-12
7	protein targeting	1.89E-12
8	cellular component organization or biogenesis	2.94E-12
9	developmental process	3.93E-12
10	multi-organism process	5.57E-12
#	Gmnn-dep H3K9ac-down ES-CNS-Networks	pValue
1	Translation_Translation initiation	6.07E-06
2	Cell cycle_S phase	1.75E-04
3	Development_Blood vessel morphogenesis	3.00E-04
4	Translation_Elongation-Termination	4.22E-04
5	Immune response_Phagocytosis	4.73E-04
6	Cell cycle_Core	1.50E-03
7	Reproduction_Spermatogenesis, motility and copulation	1.59E-03
8	Cytoskeleton_Intermediate filaments	4.54E-03
9	Reproduction_Feeding and Neurohormone signaling	1.19E-02
10	Immune response_Phagosome in antigen presentation	1.42E-02

Figure S6

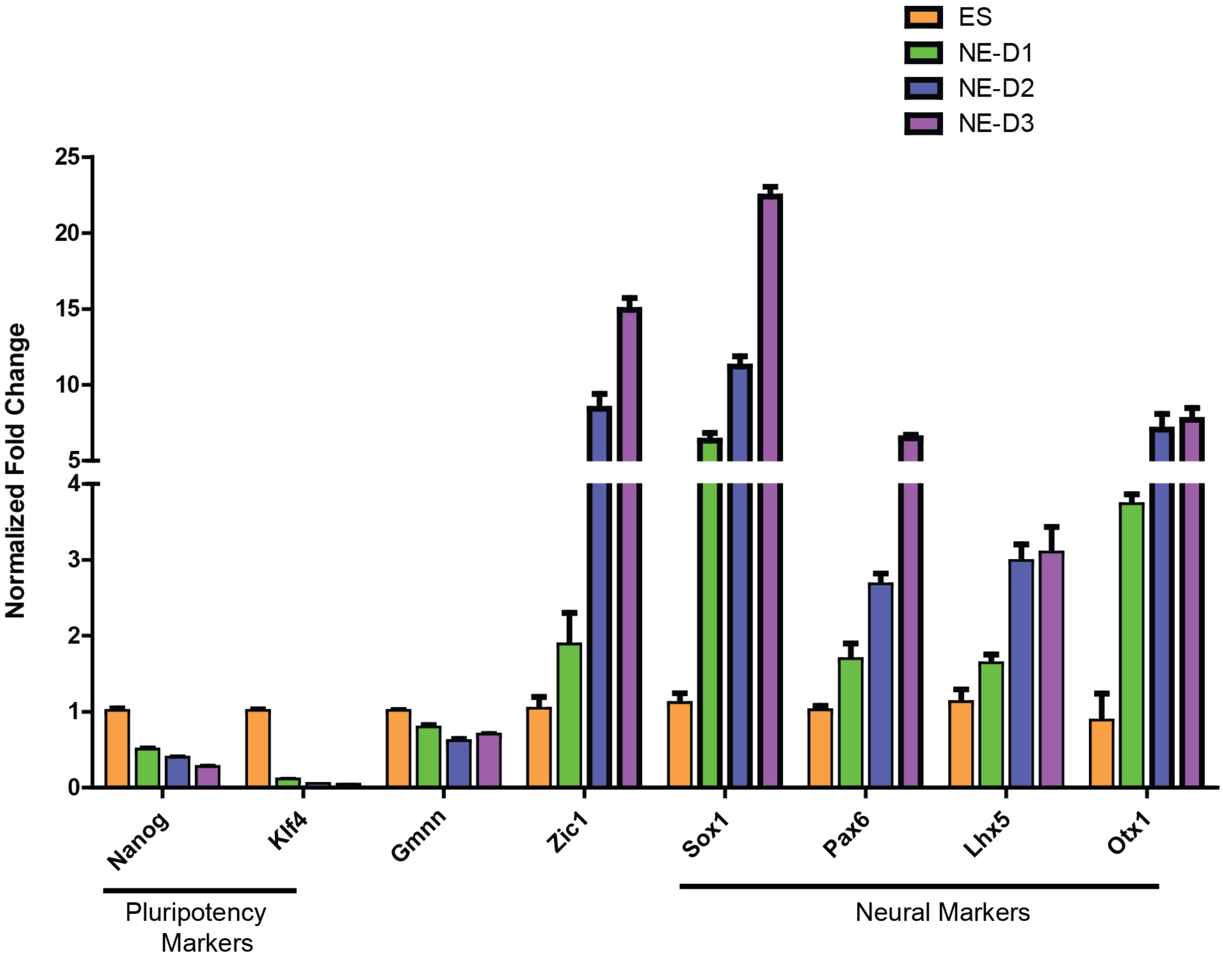
ES
NE-D1
NE-D2
NE-D3
Normalized Fold Change
25
20
15
10
5
4
3
2
1
0
Nanog
Klf4
Gmnn
Zic1
Sox1
Pax6
Lhx5
Otx1
Pluripotency Markers
Neural Markers

Figure S7

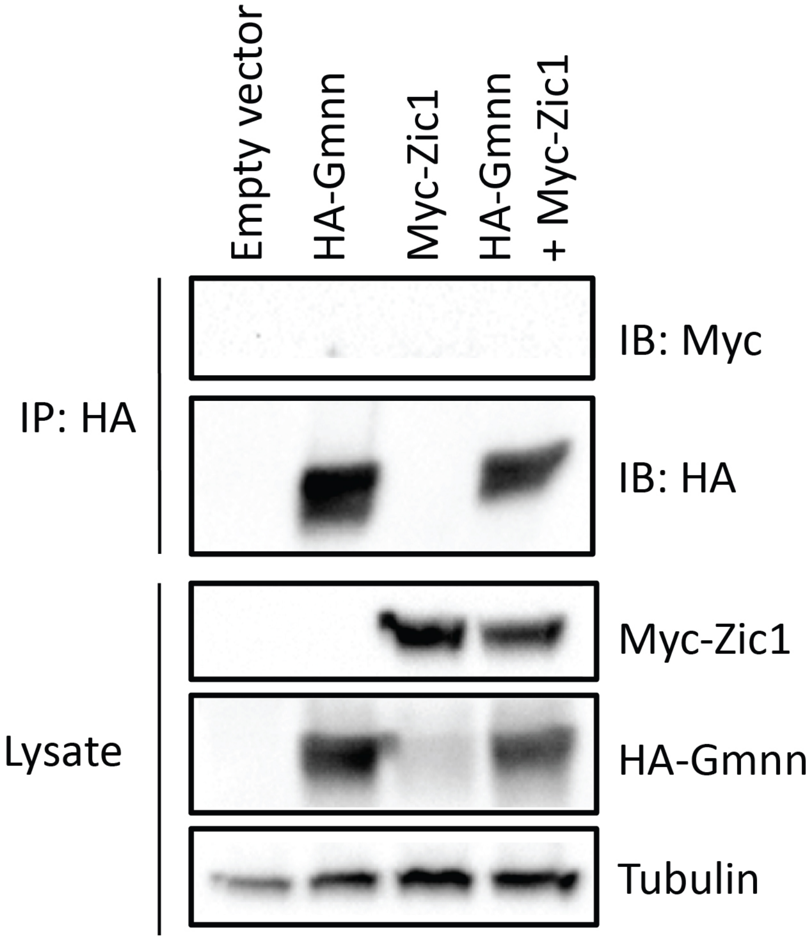
Empty vector
HA-Gmnn
Myc-Zic1
HA-Gmnn + Myc-Zic1
IP: HA
IB: Myc
IB: HA
Lysate
Myc-Zic1
HA-Gmnn
Tubulin

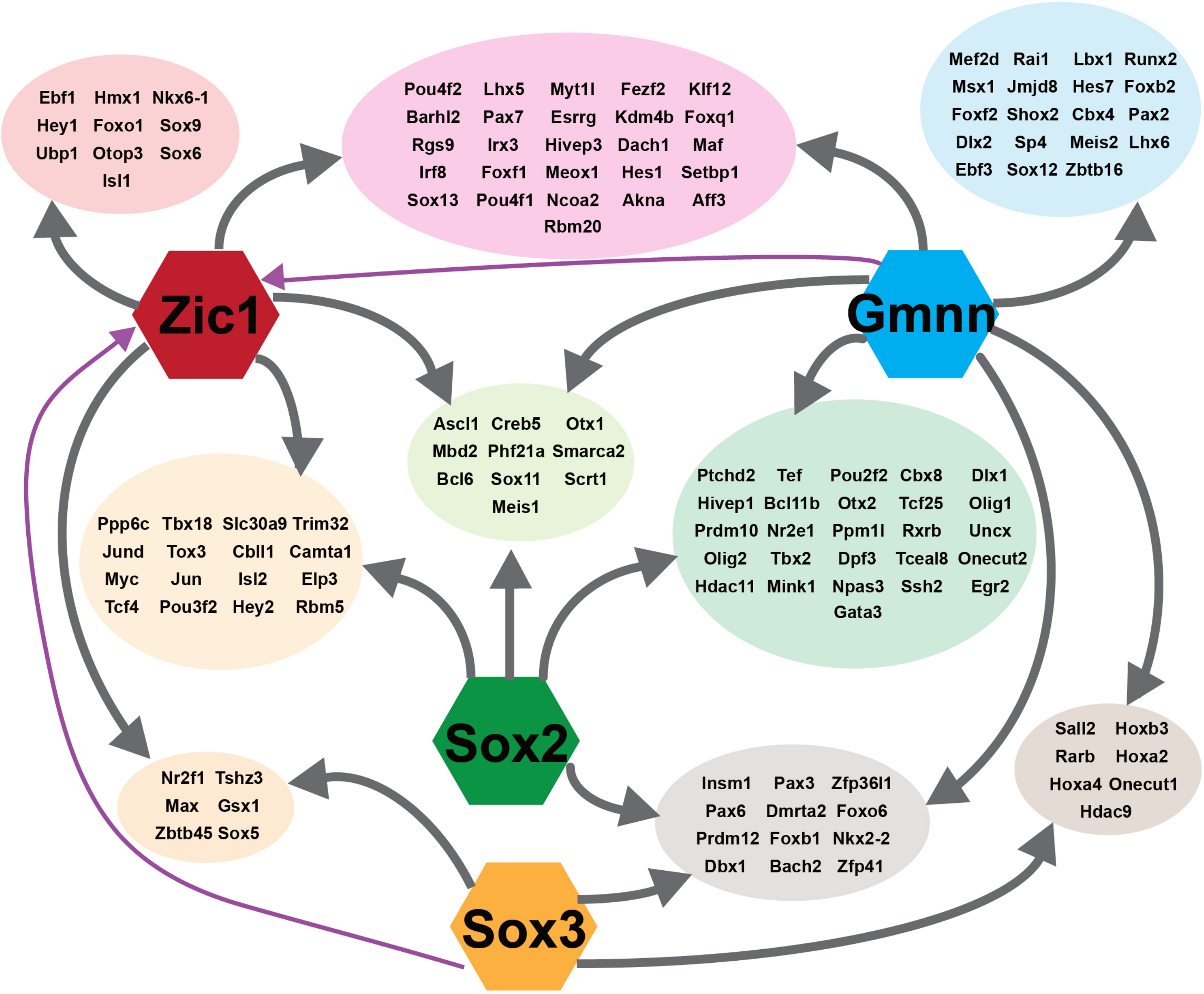
Zic1
Gmnn
Sox2
Sox3
Ebf1 Hmx1 Nkx6-1
Hey1 Foxo1 Sox9
Ubp1 Otop3 Sox6
Isl1
Pou4f2 Lhx5 Myt1l Fezf2 Klf12
Barhl2 Pax7 Esrrg Kdm4b Foxq1
Rgs9 Irx3 Hivep3 Dach1 Maf
Irf8 Foxf1 Meox1 Hes1 Setbp1
Sox13 Pou4f1 Ncoa2 Akna Aff3
Rbm20
Mef2d Rai1 Lbx1 Runx2
Msx1 Jmjd8 Hes7 Foxb2
Foxf2 Shox2 Cbx4 Pax2
Dlx2 Sp4 Meis2 Lhx6
Ebf3 Sox12 Zbtb16
Ascl1 Creb5 Otx1
Mbd2 Phf21a Smarca2
Bcl6 Sox11 Scrt1
Meis1
Ptchd2 Tef Pou2f2 Cbx8 Dlx1
Hivep1 Bcl11b Otx2 Tcf25 Olig1
Prdm10 Nr2e1 Ppm1l Rxrb Uncx
Olig2 Tbx2 Dpf3 Tceal8 Onecut2
Hdac11 Mink1 Npas3 Ssh2 Egr2
Gata3
Ppp6c Tbx18 Slc30a9 Trim32
Jund Tox3 Cbll1 Camta1
Myc Jun Isl2 Elp3
Tcf4 Pou3f2 Hey2 Rbm5
Nr2f1 Tshz3
Max Gsx1
Zbtb45 Sox5
Insm1 Pax3 Zfp36l1
Pax6 Dmrta2 Foxo6
Prdm12 Foxb1 Nkx2-2
Dbx1 Bach2 Zfp41
Sall2 Hoxb3
Rarb Hoxa2
Hoxa4 Onecut1
Hdac9
